# Effectiveness of Multi-Layer Perceptron-Based Binary Classification Neural Network in Detecting Breast Cancer Through Nine Human Serum Protein Markers

**DOI:** 10.3390/cancers17172832

**Published:** 2025-08-29

**Authors:** Eun-Gyeong Lee, Jaihong Han, Seeyoun Lee, Sung-Soo Kim, Young-Min Park, Dong-Eun Lee, Yumi Kim, Dong-Young Noh, So-Youn Jung

**Affiliations:** 1 Department of Surgery, Center of Breast Cancer, Research Institute and Hospital, National Cancer Center, Goyang 10408, Republic of Korea; bnf333@ncc.re.kr (E.-G.L.); 13154@ncc.re.kr (J.H.); seeyoun@ncc.re.kr (S.L.); 2 Manufacturing and Technology Division, Bertis Inc., Yongin 16954, Republic of Korea; sungsoo.kim@bertis.com (S.-S.K.); youngmin.park@bertis.com (Y.-M.P.); 3 Biostatics Collaboration Team, Research Core Center, Research Institute of National Cancer Center, Goyang 10408, Republic of Korea; dong-eun@ncc.re.kr; 4 Department of Surgery, CHA Gangnam Medical Center, CHA University School of Medicine, Seoul 06135, Republic of Korea; ymkim@chamc.co.kr (Y.K.); dynoh@chamc.co.kr (D.-Y.N.)

**Keywords:** nine-protein signature, proteome, breast cancer, diagnosis, deep learning

## Abstract

**Background/Objectives**: A newly developed nine-protein serum signature has been utilized to enhance the accuracy of an existing three-protein signature used as a blood-based diagnostic tool. This study used the new nine-protein serum signature to evaluate the clinical sensitivity and specificity of a medical device designed to test the clinical performance of an artificial intelligence algorithm. **Methods**: A blood-based test using multiple reaction monitoring via mass spectrometry was performed to quantify nine proteins (APOC1, CHL1, FN1, VWF, PPBP, CLU, PRDX6, PRG4, and MMP9) in serum samples from 243 healthy controls and 222 patients with breast cancer. **Results**: Based on cutoff values determined by an artificial intelligence-based deep learning model, the sensitivity and specificity of the nine-protein signature in diagnosing breast cancer among all participants was 83.3% and 88.1%, respectively, whereas those of the three-protein signature were 71.6% and 85.3%, respectively. The assay yielded a positive predictive value of 86.5% for breast cancer and 13.6% for healthy controls, with corresponding negative predictive values of 14.7% and 85.3%, respectively. The accuracies of nine- and three-protein signatures were 85.8% (area under the receiver operating characteristic curve: 0.8526) and 77.0%, respectively. **Conclusions**: The nine-protein signature may help detect breast cancer more accurately and effectively than the three-protein signature.

## Simple Summary

Breast cancer is the most common cancer among women worldwide, and current screening methods are primarily based on imaging. However, there is a growing need for a simple, minimally invasive, and cost-effective screening approach to enable early detection. The commonly used CA15-3 immunoassay has limited accuracy. In this study, a new blood-based method was developed using nine specific proteins (APOC1, CHL1, FN1, VWF, PPBP, CLU, PRDX6, PRG4, and MMP9) measured from patient serum samples. This nine-protein signature demonstrated improved diagnostic performance compared to a previous three-protein version (APOC1, CA1, and CHL1). These findings suggest that the enhanced test, supported by artificial intelligence, may help diagnose breast cancer more accurately and efficiently.

## 1. Introduction

Breast cancer is the most commonly diagnosed cancer among women worldwide and the leading cause of cancer-related mortality [1,2]. Current breast cancer screening methods are primarily based on imaging. Mammography, the primary screening tool, raises concerns owing to radiation exposure and low accuracy in detecting cancer within dense breast tissues [3]. Moreover, the procedure is uncomfortable and painful for patients due to bodily exposure and compression of their breasts [4]. Ultrasonography, although useful, is expensive and less accessible, with diagnostic outcomes potentially varying according to the operator’s skill and equipment [5,6]. Therefore, a simple, minimally invasive, and cost-effective screening method is necessary for early detection. Blood tests are one of the most suitable methods.

Although the CA15-3 immunoassay is used as a blood test for detecting breast cancer, its accuracy is low in the early stages of the disease; it is primarily used for monitoring patients undergoing treatment [7,8]. Despite the fact that contemporary proteomic profiling technologies are now capable of capturing almost the entire spectrum of human proteins, the majority of early candidates are eliminated during validation, and reproducibility remains a significant challenge [9,10]. Recently, multi-marker panels have emerged as promising alternatives for accurate diagnoses in clinical settings. For example, DNA methylation markers in peripheral blood mononuclear cells could accurately detect early-stage breast cancer through a multiplex quantitative methylation-specific PCR assay, and multi-omics integration enhances breast cancer subtyping and biomarker discovery, reinforcing the need for multi-marker panels rather than reliance on individual markers [11]. A proteomic assay was developed using the quantitative values of three biomarkers present in human plasma. It showed a diagnostic sensitivity, specificity, and accuracy of 71.6%, 85.3%, and 77.0%, respectively [12,13]. Growing interest in artificial intelligence-based diagnostic approaches has been accompanied by recent reports describing the use of artificial intelligence algorithms for breast cancer diagnosis [14]. With the advancement of marker discovery technologies, we adopted a targeted strategy starting from a library of 3,393 serum-detectable proteins. Importantly, the same analytical conditions were applied throughout both the discovery and validation phases, thereby minimizing the gap between candidate selection and clinical verification. This approach ensures analytical consistency, improves the reliability of candidate selection, and enhances efficiency across the biomarker development pipeline.

Using this methodology, we identified nine proteins—FN1, VWF, PRG4, MMP9, CLU, PRDX6, PPBP (CXCL7), APOC1, and CHL1—that were robustly quantified in serum and showed statistically significant differences between breast cancer patients and healthy controls. Building on this nine-protein signature, we developed a multilayer perceptron (MLP)-based machine learning algorithm to establish a multi-marker diagnostic model, which achieved an AUC of 0.9105. This represents a substantial improvement over conventional single-marker or limited multi-marker assays and highlights the strong potential of this panel as a clinically applicable tool for early breast cancer detection [15].

This study aimed to evaluate the clinical sensitivity and specificity of a medical device designed for testing the clinical performance of an artificial intelligence algorithm. We confirmed the clinical validity of the software that incorporates this algorithm.

## 2. Materials and Methods

### 2.1. Study Design

This multiprotein assessment, using serum samples to detect breast cancer, was part of a trial conducted in the Republic of Korea. The study was conducted by distributing the samples stored in the National Cancer Center Biobank in the Republic of Korea. The anonymization officer at the sample-providing institution replaced personal identification information with a unique identifier to ensure anonymity, whereas the randomization officer at the same institution conducted random allocation separately. Preprocessing and quantitative analyses were performed on the samples with assigned randomization numbers. The quantitative values of the serum protein markers derived from the samples were input into the software of the medical device, and the results were obtained.

We selected samples that met all the following criteria.

(1) Common selection criteria: Serum samples from adults aged 19 years or older at the time of blood collection, those stored in volumes of 200 μL or more, those that did not undergo repeated thawing and were stored in an ultra-low temperature freezer at −70 °C or below, those stored in the National Cancer Center Human Biological Material Bank, and those from individuals who had agreed to donate their samples by signing a donation consent form for human-derived materials.
(2) Patients with breast cancer: Serum samples collected before surgery from women diagnosed with breast cancer at the time of blood collection and from women with no other primary cancers besides breast cancer at the time of blood collection.
(3) Healthy controls: Serum samples from women not diagnosed with breast cancer at the time of blood collection and from women with no history of breast cancer or any other cancer diagnosis within the past 10 years at the time of blood collection.

### 2.2. Sample Size Calculation

In the exploratory clinical trial, the mean clinical sensitivity for distinguishing patients with and without breast cancer was 85.1% (95% confidence interval [CI]: 76.60–93.64), and the mean specificity was 83.5% (95% CI: 75.77–91.31) [15]. Blood samples from 213 patients with breast cancer were collected to determine clinical sensitivity, and 230 samples from the healthy controls were collected to determine clinical specificity. Considering a dropout rate of 5%, we planned to collect 225 blood samples from patients with breast cancer and 243 samples from the healthy controls.

### 2.3. Quantitative Protein Analysis

We utilized a newly developed quantitative peptide library (PepQuant library, Bertis Inc., Gwacheon-si, Republic of Korea) for additional screening of serum biomarkers [15]. We initially selected 30 markers as primary candidates from 50 cases of breast cancer and 50 healthy cases based on whether there was a fold change greater than 1.2 or less than 0.8 (*p*-value < 0.05). To ensure reproducible marker selection, we re-evaluated these primary candidates in a new set of 96 breast cancer cases and 95 healthy cases, using the same criteria. This process reduced the number of candidate markers from 30 to 16. We then tested the analytical performance of these 16 candidate markers using a mass spectrometer, ultimately identifying nine markers (APOC1, CHL1, FN1, VWF, PPBP, CLU, PRDX6, PRG4, and MMP9) that exhibited performance above the threshold, and selected them as the final markers.

Neat serum samples were trypsin-digested after reduction and alkylation, desalted using C18 cartridges, and reconstituted in 0.1% formic acid. Peptides were analyzed by LC–MRM on a QTRAP 5500 Plus (Sciex, Framingham, MA, USA) with an Agilent ZORBAX 300SB-C18 column (Agilent Technologies, Wilmington, DE, USA, 0.5 × 150 mm, 3.5 μm), using a 10-min gradient from 2.5% to 30% solvent B at a 20 μL/min flow rate. Quantification was performed using isotope-labeled internal standards (IS), and absolute concentrations were calculated from analyte-to-IS ratios. Analytical performance, including linearity, accuracy, precision, and stability, was evaluated according to established guidelines. Peptides meeting all criteria were selected for final validation.

### 2.4. Deep Learning-Based Classification Model

A supervised deep learning algorithm was developed for biomarker-based classification using a multi-layer perceptron (MLP) architecture. The MLP used in this study is a feed-forward neural network composed of an input layer, one or more hidden layers, and an output layer. Each neuron in a given layer is connected to the neurons in the subsequent layer through trainable weights, and the input features are transformed via linear combinations of these weights. Non-linear activation functions such as ReLU are then applied, allowing the network to capture complex, non-linear relationships in the data. During training, the parameters are optimized using backpropagation and gradient descent to minimize the loss function. The final output layer produces the predicted probability for classification. The model design was inspired by the GrowNet framework, which incrementally enhances feature representation through a sequential learning process. Unlike traditional single MLP models, this architecture adopts a boosting-like structure that allows progressive refinement of hidden features, thereby improving both representational capacity and generalization performance. To enhance stability and prevent overfitting, techniques such as dropout regularization and batch normalization were applied. The deep learning model was implemented in Python (v3.8.13) using the PyTorch deep learning framework (v1.7.1). Data preprocessing and manipulation were performed using NumPy (v1.24.4) and pandas (v1.4.2). Model training and inference were conducted on a GPU-enabled system with CUDA (v11.0) and cuDNN (v8005) acceleration [15].

### 2.5. Blood Sample Preparation

The serum samples were pre-treated using a standardized workflow consisting of sequential reduction, alkylation, and enzymatic digestion under controlled buffer conditions. Briefly, samples underwent denaturation and reduction at 35 °C, alkylation at room temperature in the dark, and overnight digestion at 37 °C. Reactions were then terminated with a stop buffer, and the digested mixtures were prepared using diluted MASTO-CHECK2 standard buffer. Detailed reagent compositions and incubation conditions are provided in the Supplementary Methods.

### 2.6. Estimation of the Cutoff Value

The cutoff value was set at 0.7356, which was determined as the average cutoff obtained using the index of union formula, which maximizes sensitivity and specificity across five random sets. The results obtained from the algorithm were interpreted as positive if they were equal to or greater than 0.7356 and negative if they were below this threshold.

### 2.7. Statistical Analysis

Population characteristics were summarized using the mean and standard deviation for continuous variables and frequency counts with percentages for categorical variables. The performance of diagnostic tests was assessed using statistical metrics, such as sensitivity, specificity, positive predictive value, and negative predictive value. All statistical analyses were performed using SAS (version 9.4), and a *p*-value < 0.05 was considered statistically significant. A 95% two-sided CI was computed using the exact confidence interval method.

## 3. Results

### 3.1. Quantitative Protein Analysis

Nine protein biomarkers (FN1, VWF, PRG4, MMP9, CLU, PRDX6, PPBP, APOC1, and CHL1) [16,17,18,19,20,21,22,23,24] (Table S1) were identified using samples from 187 healthy controls and 215 patients with breast cancer. Thus, 402 samples were used to train various machine learning models, with 70.0% of the combined samples allocated for training and the remaining 30.0% set aside for testing [15].

The algorithm was validated with new samples using the nine protein markers. Protein biomarker levels were measured using mass spectrometry.

### 3.2. Optimization of the Proteomic Assay

The clinical sensitivity and specificity were assessed through an exploratory clinical trial conducted to train and validate the deep learning algorithm. The sensitivity was 85.1% (95% CI: 76.60–93.64), and the average clinical specificity was 83.5% (95% CI: 75.77–91.31). These results were used to calculate the sample size required for the final confirmatory experiment [15].

### 3.3. Validation of the Proteomic Assay

To validate the optimized algorithm, 465 serum samples were obtained from 222 patients with breast cancer and 243 healthy controls.

The mean age was 53.68 ± 10.57 years at diagnosis among patients with breast cancer and 46.26 ± 9.65 years among healthy controls. The mean body mass index was 24.49 ± 3.67 and 22.11 ± 2.60 in patients with breast cancer and healthy controls, respectively. Among the breast cancer patients, stage 2 cancer was the most common with 107 patients (48.2%), followed by stage 1 with 77 patients (34.7%). There were also 23 patients (10.3%) with Stage 0 cancer (Table 1).

Using the nine-protein signature, the positive predictive value was 86.5% and 13.6% for breast cancer patients and healthy controls, respectively, while the negative predictive value was 14.7% and 85.3%, respectively (Table 2). The sensitivity was 83.3% (95% CI: 78.4–88.2%), and the specificity was 88.1% (95% CI: 84.0–92.1%). The lower limit of the CI for sensitivity, 78.4%, was 1.69 percentage points lower than that of a previous clinical trial (80.1%), thus not meeting the non-inferiority criterion. However, for specificity, the lower limit of the CI was 84.0%, which was 5.45 percentage points higher than the previously presented 78.5%, thus meeting the non-inferiority criterion (Table 3). The sensitivity and specificity by cancer stage were as follows. For stage 0, the sensitivity and specificity were 73.9% and 88.1%, respectively. For stage 1, the sensitivity was 87.0% and specificity was 88.1%. For stages 2 and 3, the sensitivity and specificity were 75.4% and 88.1%, respectively, and 80.0% and 88.07%, respectively.

**Figure 1:**
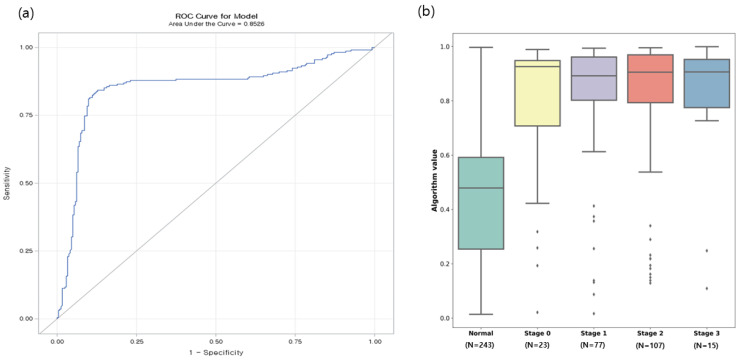
Breast cancer prediction accuracy. (**a**) Receiver operating characteristic (ROC) graph illustrating the area under the curve for the proteomic assay’s diagnostic performance. (**b**) Box plot displaying the predicted probability distribution of breast cancer across different stages.

The diagnostic accuracy of the proteomic assay remained robust, as demonstrated by an area under the curve of 0.8526 (Figure 1a). To gain additional insight into the performance of the model, we visualized the distribution of the predicted probabilities for different stages of breast cancer in the test data. The model showed a similar prediction pattern for early- and advanced-stage breast cancer (*p* < 0.0001) (Figure 1b). In addition to the AUC, we evaluated model performance using Accuracy and F1-score. The proposed model achieved an Accuracy of 93.4% and an F1-score of 0.9107, which further supports its robustness compared with conventional approaches. Among all models, MastoCheck2 showed the most robust performance, achieving perfect specificity (1.0000), the highest accuracy (0.9338), and the best F1-score. (Table 4).

## 4. Discussion

This study investigated the precision of blood-based proteomic biomarkers for breast cancer diagnosis using multiple reaction monitoring via liquid chromatography–tandem mass spectrometry optimized for a multi-marker assay. Nine biomarkers (APOC1, CHL1, FN1, VWF, PPBP, CLU, PRDX6, PRG4, and MMP9) in human serum were quantitatively analyzed using mass spectrometry, and the data were subsequently input into a novel deep-learning algorithm to detect breast cancer.

Mammography, which is widely used as the standard screening approach, achieves sensitivity estimates of 77.0–95.0% in the general population. Its diagnostic accuracy, however, is significantly reduced in women with dense breasts, with sensitivity falling to 30.0–48.0%, while specificity is consistently reported at 94.0–97.0% [25,26].

In a previous multicenter, prospective trial, multiple reaction monitoring-mass spectrometry was used to analyze blood samples from participants with the aim of improving early breast cancer detection, particularly in women with dense breast tissue. Three marker proteins APOC1, CA1, and CHL1 were considered [13]. The overall sensitivity of this biomarker test is 74.4%, with moderate specificity around 60.9–66.9%, indicating its potential usefulness in clinical settings [27].

Serum is often preferred in clinical settings owing to its ease of use and stability. It is relatively easy to handle, and its stable nature reduces the risk of degradation, allowing for long-term storage. Therefore, serum is widely used in various clinical experiments, including biomarker tests [28,29,30].

A peptide library, PepQuant, was designed for discovering and validating blood biomarkers indicating breast cancer using mass spectrometry. The library consists of 852 quantifiable peptides representing 452 blood proteins. In the present study, 30 potential biomarkers for breast cancer were identified, of which nine were validated. A machine learning model that incorporated these biomarkers has demonstrated high accuracy in predicting breast cancer, with an area under the curve of 0.9105 [15]. The biomarkers validated in the present study were shown to work well with this machine learning model, suggesting that they may enhance early detection when used alongside current breast cancer screening methods.

However, this study has several limitations. First, the blood samples were collected from a single institution. To address this issue, we conducted a pilot study. When using the serum of 30 healthy women and 30 patients with breast cancer to diagnose breast cancer, the nine-protein signature showed a sensitivity and specificity of 87.9% and 80.7%, respectively (Table S2). These values were 20% and 10% higher than those of the three-protein signature, which had a sensitivity and specificity of 66.7% and 70.0%, respectively. The accuracies of the nine- and three-protein signatures were 83.3% and 68.3%, respectively. We then expanded the sample size in the current study to obtain more conclusive findings based on the results of the pilot study. Second, we used samples stored in a biobank. Therefore, our study is a retrospective study, which is susceptible to the inherent limitations of the study design. In our study, the clinical sensitivity was 83.3% (95% CI: 78.4–88.2%) and the specificity was 88.1% (95% CI: 84.0–92.1%). According to the performance criteria for sensitivity, the lower limit of the CI should be above 80.1%; however, in this case, it was 78.4%, falling short by 1.7 percentage points. In contrast, the specificity exceeded our expectations by 5.5 percentage points. This slight reduction in sensitivity may be attributed to the exploratory nature of the clinical validation of the algorithm. The sensitivity for stage 0 disease was relatively lower (73.9%) compared with invasive stages. This may be partly explained by the limited number of stage 0 cases, which reduces statistical stability. Nevertheless, given that early detection remains the primary clinical goal, further validation with larger stage 0 cohorts is warranted. We anticipate that increasing the sample size in future prospective studies will yield improved results. Therefore, a large-scale prospective study is required to properly assess the efficacy of this assay.

## 5. Conclusions

The nine-protein signature may be a more accurate and effective tool for detecting breast cancer than the three-protein signature, offering a broader range of biomarkers that could potentially increase the sensitivity and specificity of a serum-based diagnostic tool. The enhanced detection capability of the nine-protein signature may enable earlier and more reliable diagnosis.

## Figures and Tables

**Table 1:** Clinicopathologic characteristics of the study population.

Characteristics	Patients with Breast Cancer (N = 222, %) ^(a)^	Healthy Control (N = 243, %) ^(a)^
Age (y)	53.68 ± 10.57	46.26 ± 9.65
Height (cm)	158.38 ± 5.90	160.53 ± 4.88
Weight (kg)	61.37 ± 9.49	57.00 ± 7.21
BMI (kg/m^2^)	24.49 ± 3.67	22.11 ± 2.60
AJCC stage		
0	23 (10.3)	
1	77 (34.7)	
2	107 (48.2)	
3	15 (6.8)	
4	0 (0)	

^(a)^ Values are presented as mean ± standard deviation or number (%). BMI, body mass index; AJCC, American Joint Committee on Cancer.

**Table 2:** Predictive value among the population using the nine-protein signature.

	Patients with Breast Cancer (N = 222, %)	Healthy Control (N = 243, %)	ALL (N = 465, %)
Positive predictive value	185 (86.5)	29 (13.6)	214 (46.0)
Negative predictive value	37 (14.7)	214 (85.3)	251 (54.0)
Total	222 (47.7)	243 (52.3)	465(100.0)

**Table 3:** Diagnostic accuracy of the nine-protein signature.

	Sensitivity	Specificity
Value	0.8333	0.8807
95% Confidence interval	0.7843–0.8824	0.8399–0.9214

**Table 4:** Classification performance of different models evaluated on an internal validation set, generated by randomly partitioning approximately 30% (151/502) of the development cohort into an independent test set. Confirmatory clinical data were not used.

Model	Sensitivity	Specificity	AUC	Accuracy	F1-Score
Logistic Regression	0.8197	0.9667	0.9554	0.9073	0.8772
SVM (RBF Kernel)	0.8033	0.9667	0.9541	0.9007	0.8673
K-Nearest Neighbors	0.8525	0.9667	0.9510	0.9205	0.8966
Gradient Boosting	0.8689	0.9667	0.9876	0.9272	0.9060
MastoCheck2	0.8361	1.0000	0.9770	0.9338	0.9107

## Data Availability

The datasets used and analyzed during the current study are available from the corresponding author on reasonable request.

## Supplementary Materials

The following supporting information can be downloaded at: https://www.mdpi.com/article/10.3390/cancers17172832/s1, Table S1: Function of nine proteins in tumors and the tumor microenvironment [19,23,31,32,33,34,35,36,37]; Table S2: Sensitivity, specificity, and accuracy of the 3-protein signature and nine-protein signature in the pilot study.

## Abbreviations

The following abbreviations are used in this manuscript:

CI	Confidence interval
ROC	Receiver operating characteristic
