# Supplementary material for: Effectiveness of Multi-Layer Perceptron-Based Binary Classification Neural Network in Detecting Breast Cancer Through Nine Human Serum Protein Markers
Source: Cancers (Basel). 2025 Aug 29;17(17):2832. doi: 10.3390/cancers17172832 (PMC12427305; doi:10.3390/cancers17172832)
Supplement: Supplementary file 1 [file cancers-17-02832-s001.zip › cancers-3815983-supplementary.pdf]

Effectiveness of Multi-Layer Perceptron-Based Binary Classification Neural Network in Detecting Breast Cancer Through Nine Human Serum Protein Markers

Supplementary Methods

Blood sample preparation

The samples were pre-treated using the following protocol. First, 0.75 mL reduction buffer was mixed with 6.75 mL denaturing buffer. Then, 75 μL of this solution was dispensed into each well of a 96-well plate, to which 5 μL of serum sample was added and mixed. Subsequently, the mixture was incubated at 35°C for 1 h and 30 min. Alkylation buffer was then cooled to room temperature, added at 7.5 μL to each well, and mixed thoroughly with the contents of the well. The mixture was then incubated in the dark at room temperature for 30 min. Thereafter, 600 μL of a solution consisting of 10 μL digestion buffer mixed with 590 μL dilution buffer was dispensed into each well. The 96-well plate was covered with an evaporation-prevention cover and incubated at 37°C for 16 h. Subsequently, 65 μL reaction stop buffer was added to each well and mixed thoroughly. The sample was further mixed with 100-fold diluted MASTO-CHECK2 standard buffer.

Table S1. Function of nine proteins in tumors and the tumor microenvironment

Marker	Functional Role in Breast Cancer	Reference (Full Title)	Cancer Hallmarks (Hanahan & Weinberg, Cell 2000, 2011 update)
**MMP9[31]**	MMP-9 cooperates with transcription factor Snail to drive epithelial–mesenchymal transition (EMT), promoting tumor cell invasion and metastatic potential by enhancing EMT-associated pathways.	Lin CY, Tsai PH, Kandaswami CC, Lee PP, Huang CJ, Hwang JJ, Lee MT. Matrix metalloproteinase-9 cooperates with transcription factor Snail to induce epithelial-mesenchymal transition. Cancer Sci. 2011 Apr;102(4):815-27. doi: 10.1111/j.1349-7006.2011.01861.x. Epub 2011 Feb 9. PMID: 21219539.	Activating invasion and metastasis

**PRDX6**[32]	Downregulation of PRDX6 via RNA interference reduces breast cancer cell invasion and metastasis in vitro and in vivo. Specifically, PRDX6 overexpression accelerates tumor growth and pulmonary metastasis, while knockdown suppresses these effects.	Chang XZ, Li DQ, Hou YF, Wu J, Lu JS, Di GH, Jin W, Ou ZL, Shen ZZ, Shao ZM. Identification of the functional role of peroxiredoxin 6 in the progression of breast cancer. Breast Cancer Res. 2007;9(6):R76. doi: 10.1186/bcr1789. Erratum in: Breast Cancer Res. 2018 Jul 2;20(1):63. doi: 10.1186/s13058-018-0984-0. PMID: 17980029; PMCID: PMC2246172.	Resisting cell death, Deregulating cellular energetics
**CLU**[33]	Elevated serum clusterin (sCLU) levels in invasive breast cancer patients correlate with higher tumor stage, lymph node metastasis, poorer overall and disease-free survival, and reduced chemotherapy responsiveness—suggesting sCLU as a prognostic and therapeutic response biomarker.	Chen QF, Chang L, Su Q, Zhao Y, Kong B. Clinical importance of serum secreted clusterin in predicting invasive breast cancer and treatment responses. Bioengineered. 2021 Dec;12(1):278-285. doi: 10.1080/21655979.2020.1868732. PMID: 33356806; PMCID: PMC8806267.	Resisting cell death, Tumor-promoting inflammation
**PRG4**[34]	Proteoglycans like PRG4 modulate tumor microenvironment by influencing inflammation, angiogenesis, cell adhesion, migration, and cancer metastasis through altered expression in tumor and stromal cells.	Theocharis AD, Skandalis SS, Neill T, Multhaupt HA, Hubo M, Frey H, Gopal S, Gomes A, Afratis N, Lim HC, Couchman JR, Filmus J, Sanderson RD, Schaefer L, Iozzo RV, Karamanos NK. Insights into the key roles of proteoglycans in breast cancer biology and translational medicine. Biochim Biophys Acta. 2015 Apr;1855(2):276-300. doi: 10.1016/j.bbcan.2015.03.006. Epub 2015 Mar 28. PMID: 25829250; PMCID: PMC4433619.	Inducing angiogenesis, Tumor-promoting inflammation
**FN1**[35]	Fibronectin (FN1) is a key ECM glycoprotein that enhances tumor cell attachment, spreading, invasion, and metastasis via integrin β-5 and Src-mediated signaling—especially in 3D tumor environments.	Ylätupa S, Haglund C, Mertaniemi P, Vahtera E, Partanen P. Cellular fibronectin in serum and plasma: a potential new tumour marker? Br J Cancer. 1995 Mar;71(3):578-82. doi: 10.1038/bjc.1995.112. PMID: 7880741; PMCID: PMC2033647.	Activating invasion and metastasis
**vWF**[36]	Elevated plasma vWF levels in breast cancer patients—particularly those with metastatic disease—facilitate platelet–tumor cell interactions and hematogenous dissemination,	Goh CY, Patmore S, Smolenski A, Howard J, Evans S, O'Sullivan J, McCann A. The role of von Willebrand factor in breast cancer metastasis. Transl Oncol. 2021 Apr;14(4):101033. doi: 10.1016/j.tranon.2021.101033.	Activating invasion and metastasis, Inducing angiogenesis

	thereby contributing to metastatic spread.	Epub 2021 Feb 8. PMID: 33571850; PMCID: PMC7876567.	
**PPBP (CXCL7)**[37]	CXCL7, secreted by tumor-infiltrating monocytes, promotes breast cancer cell migration, invasion, and metastasis via mechanisms involving FAK activation and MMP-13 induction. Elevated CXCL7 correlates with poor patient survival.	Wang YH, Shen CY, Lin SC, Kuo WH, Kuo YT, Hsu YL, Wang WC, Lin KT, Wang LH. Monocytes secrete CXCL7 to promote breast cancer progression. Cell Death Dis. 2021 Nov 17;12(12):1090. doi: 10.1038/s41419-021-04231-4. PMID: 34789744; PMCID: PMC8599470.	Tumor-promoting inflammation, Activating invasion and metastasis
**APOC1**[38]	ApoC-I peptides are reduced in breast cancer and functionally inhibit cancer cell proliferation and tumor growth—indicating both diagnostic and therapeutic potential.	Sun Y, Zhang J, Guo F, Zhao W, Zhan Y, Liu C, Fan Y, Wang J. Identification of Apolipoprotein C-I Peptides as a Potential Biomarker and its Biological Roles in Breast Cancer. Med Sci Monit. 2016 Apr 7;22:1152-60. doi: 10.12659/msm.896531. PMID: 27052600; PMCID: PMC4827518.	Deregulating cellular energetics
**CHL1**[39]	CHL1 expression is silenced by promoter hypermethylation in breast cancer, which serves as an independent prognostic factor—downregulation of CHL1 (due to hypermethylation) is linked to poor outcomes.	Martín-Sánchez E, Mendaza S, Ulazia-Garmendia A, Monreal-Santesteban I, Blanco-Luquin I, Córdoba A, Vicente-García F, Pérez-Janices N, Escors D, Megías D, López-Serra P, Esteller M, Illarramendi JJ, Guerrero-Setas D. CHL1 hypermethylation as a potential biomarker of poor prognosis in breast cancer. Oncotarget. 2017 Feb 28;8(9):15789-15801. doi: 10.18632/oncotarget.15004. PMID: 28178655; PMCID: PMC5362523.	Activating invasion and metastasis, Evading growth suppressors

Table S2. Sensitivity, specificity, and accuracy of the 3-protein signature and nine-protein signature in the pilot study

	9-protein signature	3-protein signature
Sensitivity	87.9%	71.6%
Specificity	80.7%	85.3%
Accuracy	83.3%	77.0%

References

31. Lin, C.Y.; Tsai, P.H.; Kandaswami, C.C.; Lee, P.P.; Huang, C.J.; Hwang, J.J.; Lee, M.T. Matrix metalloproteinase-9 cooperates with transcription factor Snail to induce epithelial–mesenchymal transition. *Cancer science* **2011**, *102*, 815-827.
32. Chang, X.-Z.; Li, D.-Q.; Hou, Y.-F.; Wu, J.; Lu, J.-S.; Di, G.-H.; Jin, W.; Ou, Z.-L.; Shen, Z.-Z.; Shao, Z.-M. Identification of the functional role of peroxiredoxin 6 in the progression of breast cancer. *Breast Cancer Research* **2007**, *9*, R76.
33. Chen, Q.-F.; Chang, L.; Su, Q.; Zhao, Y.; Kong, B. Clinical importance of serum secreted clusterin in predicting invasive breast cancer and treatment responses. *Bioengineered* **2021**, *12*, 278-285.
34. Theocharis, A.D.; Skandalis, S.S.; Neill, T.; Multhaupt, H.A.; Hubo, M.; Frey, H.; Gopal, S.; Gomes, A.; Afratis, N.; Lim, H.C. Insights into the key roles of proteoglycans in breast cancer biology and translational medicine. *Biochimica et Biophysica Acta (BBA)-Reviews on Cancer* **2015**, *1855*, 276-300.
35. Ylätupa, S.; Haglund, C.; Mertaniemi, P.; Vahtera, E.; Partanen, P. Cellular fibronectin in serum and plasma: a potential new tumour marker? *British journal of cancer* **1995**, *71*, 578-582.
36. Goh, C.Y.; Patmore, S.; Smolenski, A.; Howard, J.; Evans, S.; O'Sullivan, J.; McCann, A. The role of von Willebrand factor in breast cancer metastasis. *Translational Oncology* **2021**, *14*, 101033.
37. Wang, Y.-H.; Shen, C.-Y.; Lin, S.-C.; Kuo, W.-H.; Kuo, Y.-T.; Hsu, Y.-L.; Wang, W.-C.; Lin, K.-T.; Wang, L.-H. Monocytes secrete CXCL7 to promote breast cancer progression. *Cell death & disease* **2021**, *12*, 1090.
38. Sun, Y.; Zhang, J.; Guo, F.; Zhao, W.; Zhan, Y.; Liu, C.; Fan, Y.; Wang, J. Identification of apolipoprotein CI peptides as a potential biomarker and its biological roles in breast cancer. *Medical science monitor: international medical journal of experimental and clinical research* **2016**, *22*, 1152.
39. Martín-Sánchez, E.; Mendaza, S.; Ulazia-Garmendia, A.; Monreal-Santesteban, I.; Blanco-Luquin, I.; Córdoba, A.; Vicente-García, F.; Pérez-Janices, N.; Escors, D.; Megías, D. CHL1 hypermethylation as a potential biomarker of poor prognosis in breast cancer. *Oncotarget* **2017**, *8*, 15789.
